# Platelet-derived circFAM13B associated with anti-platelet responsiveness of ticagrelor in patients with acute coronary syndrome

**DOI:** 10.1186/s12959-024-00620-9

**Published:** 2024-06-21

**Authors:** Yuting Zou, Yuyan Wang, Yanzhu Yao, Yangxun Wu, Chao Lv, Tong Yin

**Affiliations:** 1 Institute of Geriatrics, National Clinical Research Center for Geriatric Diseases, 2nd Medical Center, Chinese PLA General Hospital, Beijing, China; 2 Senior Department of Cardiology, The 6th Medical Center, Chinese PLA General Hospital, Beijing, China

**Keywords:** Platelet, circFAM13B, Anti-platelet efficacy, Ticagrelor, Acute coronary syndrome

## Abstract

**Background:**

Platelet is enriched with Circular RNAs (circRNAs), with circFAM13B rank among the 10 most abundant circRNAs in platelets. The aim of the present study was to evaluate the predictive value of platelet-derived circFAM13B for the antiplatelet responsiveness and efficacy of ticagrelor in patients with acute coronary syndrome (ACS).

**Methods:**

Consecutive ACS patients treated with ticagrelor were enrolled, and the antiplatelet responsiveness of 3 days of ticagrelor maintenance treatment was assessed by measuring the adenosine diphosphate (ADP)-induced platelet inhibition rate (ADP%) using thromboelastography. The expression of circFAM13B in the patients’ platelets was analyzed by quantitative real-time polymerase chain reaction. The correlation between circFAM13B expression and ticagrelor antiplatelet responsiveness, as well as the independent contribution of circFAM13B to the composite of adverse ischemic events during a follow-up period of at least 12 months was evaluated.

**Results:**

A total of 129 eligible ACS patients treated with ticagrelor were enrolled in the study. A negative correlation was found between the expression of circFAM13B and the ADP% value (*r* = -0.41, *P* < 0.001). Patients with ADP% ≥ 76% had a significantly lower level of circFAM13B compared to those with ADP% < 76% (adjusted *P* = 0.009). Receiver operating characteristic curve analysis demonstrated that combining circFAM13B expression > 1.05 with clinical risk factors could effectively predict the risk of adverse ischemic events (AUC = 0.81, 95% CI: 0.69 to 0.92, *P* < 0.001). Kaplan-Meier survival analysis showed that patients with circFAM13B > 1.05 had a significantly higher risk of adverse ischemic events compared to those with circFAM13B ≤ 1.05 (*P* = 0.003). Multivariate logistic hazard analysis identified circFAM13B > 1.05 as an independent risk factor for adverse ischemic events in in ticagrelor-treated ACS patients (adjusted OR: 5.60, 95% CI: 1.69–18.50; *P* = 0.005).

**Conclusions:**

Platelet-derived circFAM13B could be utilized for predicting the antiplatelet responsiveness and efficacy of ticagrelor in patients with ACS.

## Introduction

Acute coronary syndrome (ACS) is the most prevalent and fatal clinical manifestation of cardiovascular disease. It is widely acknowledged that platelet aggregation plays an essential role in the pathogenesis of ACS. Dual antiplatelet therapy (DAPT) with a P2Y12 receptor inhibitor and aspirin is the standard treatment for patients with ACS. Ticagrelor, as the first reversible oral P2Y12 inhibitor with more potent antiplatelet effects than clopidogrel, is recommended for the treatment of patients with ACS [1–3]. Despite the widely recognized benefit of ticagrelor, there is still variation in anti-platelet responsiveness and residual risk of ischemic events in ACS patients treated with ticagrelor [4–7]. Therefore, how to predict the occurrence of adverse events in ticagrelor-treated patients is crucial for personalized antiplatelet therapy in patients with ACS.

Platelets, as the main effectors of hemostasis and thrombosis, have been found to have an abundance of non-coding RNAs (ncRNAs) [8, 9]. Compared to other hematopoietic cell types, platelets show a higher proportion of circular RNAs (circRNAs), attributed to the general degradation of linear RNAs during the lifetime of platelets [10]. CircRNAs are generated through a unique mode of alternative splicing of pre-mRNAs, which are much more suitable as biomarkers due to the higher biological stability with the circular configuration and resistance to exoribonucleolytic degradation [10–12]. CircFAM13B was one of the 10 most abundant circRNAs predicted in platelets [11]. As a 331 bp circular RNA localized to chromosome 5, apart from platelets, circFAM13B is highly expressed in the testis, endometrium, as well as testis, endometrium [13]. Recent studies found the dysregulation of circFAM13B expression had the potential value as a biomarker for the treatment of cancer, including the inhibition of bladder cancer proliferation by attenuating the glycolytic process, and acting as a molecular sponge for miR-212 in hepatocellular carcinoma [14–16]. However, as one of the highly abundant circRNAs in human platelets, the role of circFAM13B has never been investigated in the platelets. The aim of the present study was to evaluate the predictive value of platelet-derived circFAM13B in relation to the antiplatelet efficacy of ticagrelor in patients with ACS.

## Materials and methods

### Patient recruitment

In the study, patients with ACS treated by ticagrelor for at least 1 year were consecutively recruited from April 2020 to December 2021 in the Department of Cardiology, Chinese PLA General Hospital. All the enrolled patients were treated with ticagrelor (loading dose of 180 mg or maintenance dose of 90 mg twice daily, or both) after admission to the hospital. Patients were excluded from the study if they were under 18 years old, had a history of hematologic diseases or a tendency to bleed within the last three months, a known contraindication to ticagrelor treatment, a history of serious surgery and deep puncture wounds, or severe hepatic and renal dysfunction with a life expectancy of less than 1 year. The study was approved by the institutional ethics committee of the Chinese PLA General Hospital (S2021-664-02) and conducted in accordance with the declaration of Helsinki. All enrolled patients signed informed consent.

### Measurement of ticagrelor antiplatelet responsiveness

The antiplatelet responsiveness of ticagrelor was measured for each patient using the Thromboelastography (TEG) Hemostasis Analyzer (Lepu Medical Technology, Beijing) with platelet mapping analysis. Blood samples were obtained via peripheral venipuncture after 3 days of maintenance treatment with ticagrelor, and were detected within 2 h according to the manufacturer’s instructions. The TEG system measures the maximum amplitude (MA), which is a direct parameter that reflects the maximum clot strength. The TEG system is capable of measuring thrombin-induced platelet fibrin clot strength (MA_thrombin_) and ADP-induced platelet fibrin clot strength (MA_ADP_), as well as MA generated by fibrin (MA_fibrin_). The ticagrelor antiplatelet responsiveness was evaluated according to the calculation of the ADP-induced platelet inhibition rate (ADP%) as the following formula: ADP% = [1-(MA_ADP_–MA_fibrin_)/ (MA_thrombin_– MA _fibrin_)] × 100% [17–19].

### The expression of circFAM13B in platelet

Platelets were isolated from all enrolled patients. The whole blood samples were drawn in a sodium citrate anticoagulated vacuum blood collection tube and centrifuged at 100×g for 15 min at room temperature. And 700 µl of the upper plasma layer was taken with an RNAase-free and bacteria-free tip, which was the platelet-rich plasma (PRP). The PRP was transferred into a new uncontaminated 1.5 ml EP tube and centrifuged at 2000 × g for 15 min at room temperature to remove the supernatant, and the precipitate in the tube was platelets. The expression of the circFAM13B was measured in platelets. The total RNA isolation was carried out using the high-purity total RNA isolation kit (# R1002, SinoGene, China) according to the manufacturer’s instructions. The RNA samples were then subjected to the preparation of cDNA samples using the Thermo First cDNA Synthesis Kit (# Q1010, SinoGene, China). With GAPDH serving as the internal reference, cDNA samples were used in qPCR to measure the relative expression levels of circFAM13B. qRT-PCR was performed by 2×SG Green qPCR Mix (with ROX) (# Q1002, SinoGene, China) with the standard protocol on StepOnePLUS (Applied Biosystems, USA). All experiments were performed on three biological replicates. Ct thresholds were determined by the software. The expression of circFAM13B was normalized using the 2^-ΔΔct^ method. Primer sequences were as follows: has-circ- 0001535 forward, 5′-CAATGAAGCTATGCAGCAAGA-3′ and reverse, 5′-CAAAAAGGTGCTGTTCCACA-3′; GADPH forward, 5′-GAAGGTGAAGGTCGGAGTC-3′ and reverse, 5′- GAAGATGGTGATGGGATTTC-3′. PCR thermal conditions were as follows: 10 min at 95 °C and then 40 cycles of 20 s at 95 °C and 30s at 60 °C.

### Endpoints and follow-up

The primary endpoint was the antiplatelet responsiveness of post 3 days of maintenance treatment of ticagrelor measured by TEG with the value of ADP% lower than 76%. The cut-off value was identified as reflective of high on-treatment platelet reactivity (HTPR) under ticagrelor treatment and to predict adverse ischemic events [17]. The secondary endpoints were adverse ischemic events defined as all-cause death, nonfatal myocardial infarction (MI), nonfatal stroke, stent thrombosis and rehospitalization for unstable angina (UA). Patients were followed up for at least 12 months after discharge to identify the occurrence of adverse ischemic events.

### Statistical analysis

Categorical variables are expressed as n (%) and were compared using the χ2 test. The Kolmogorov–Smirnov test was used to check for the normal distribution of continuous data. Continuous variables expressed as the mean ± standard deviation (SD) or median (interquartile range [IQR]) were compared using the t-test or Mann‒Whitney U test based on their distributions. The Pearson correlation test was utilized to evaluate the correlation between the expression level of circFAM13B and the ADP% measured by TEG. Univariate and multivariate logistic regression analysis was carried out to determine the independent predictive capability of the circFAM13B on the primary and secondary endpoints. Multivariate logistic regression analysis was adjusted for age, smoking, history of stroke, diabetes mellitus, prior percutaneous coronary intervention (PCI) and 3-vessel diseases of coronary artery. The role of circFAM13B in predicting adverse ischemic events was analyzed by performing ROC curve analysis. Kaplan–Meier estimates of adverse ischemic events were used to construct time-to-event curves according to the optimal cut-off level (relative expression of circFAM13B > 1.05). All statistical analysis was performed by the software of SPSS Statistics 22.0 (SPSS, Inc., Chicago, IL, USA), R (version 3.6.0) and GraphPad Prism 8. All p values were two-sided, with the P value < 0.05 as statistically significant.

## Results

### Patient characteristics

The basic clinical characteristics of the consecutively recruited 129 ACS patients treated with ticagrelor enrolled for the study were summarized in Table 1. It showed that the mean age of the enrolled patients was 60.76 ± 7.68 years old. According to the inclusion and exclusion criteria, 15 (11.63%) patients with STEMI, 15 (11.63%) patients with NSTEMI, and 99 (76.74%) patients with UA were finally included in the analysis. The proportion of ACS patients undergoing PCI was 97.67% in the whole study.

**Table 1:** Baseline clinical characteristics of the patients with ACS

Clinical characteristics		Total (*n* = 129)
Age, (mean ± SD) y		60.76 ± 7.68
Male, n (%)		104 (80.62)
BMI, (mean ± SD) kg/m²		25.60 ± 3.25
Smoking, n (%)		61 (47.29)
Drinking, n (%)		62 (48.06)
Hypertension, n (%)		91 (70.54)
Hyperlipidemia, n (%)		34 (26.36)
Diabetes mellitus, n (%)		44 (34.11)
Prior MI, n (%)		25 (19.38)
Prior PCI, n (%)		44 (34.11)
History of stroke, n (%)		15 (11.63)
Clinical diagnosis		
STEMI, n (%)		15 (11.63)
NSTEMI, n (%)		15 (11.63)
UA, n (%)		99 (76.74)
Undergoing PCI, n (%)		126 (97.67)
Number of diseased vessels for PCI		
1-vessel, n (%)		21 (16.28)
2-vessel, n (%)		42 (32.56)
3-vessel, n (%)	61 (47.29)	
LVEF, median (IQR) %		61 (56,64)
History of antiplatelet medication changes		
Switched from clopidogrel to ticagrelor		24 (18.60)
Switched from aspirin to ticagrelor		4 (3.10)
Concomitant medication		
ACEI/ARB, n (%)		55 (42.64)
β-Blockers, n (%)		81 (62.79)
CCB, n (%)		38 (29.46)
PPI, n (%)		81(62.79)
Statins, n (%)		124 (96.12)

*Abbreviations* ACEI, angiotensin-converting enzyme inhibitor; ARB, angiotensin receptor blocker; BMI, body mass index; BUN, blood urea nitrogen; CCB, calcium channel blocker; MI, myocardial infarction; NSTEMI, Non-ST segment elevation myocardial infarction; PCI, percutaneous coronary intervention; PPI, proton pump inhibitor; STEMI, ST-segment elevation myocardial infarction, UA, unstable angina

### The ADP-induced platelet inhibition rate measured by TEG

**Fig. 1:**
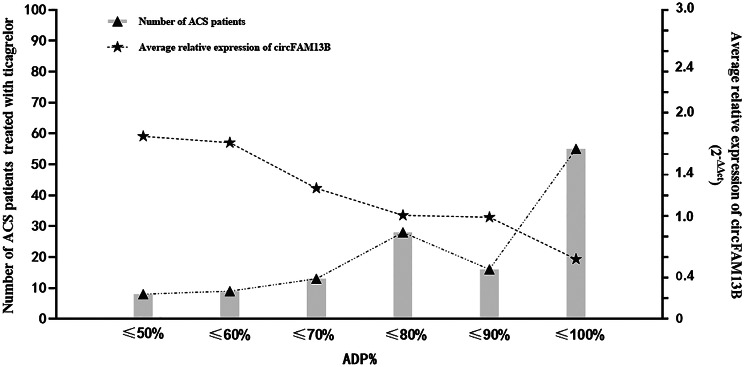
Bar charts summarized the average relative expression of circFAM13B presenting with ADP% distribution in ticagrelor-treated patients with ACS. ADP%, adenosine diphosphate (ADP)-induced platelet inhibition rate; ACS, acute coronary syndrome

The majority of ACS patients have great responsiveness to 3 days of treatment with ticagrelor, with ADP% values measured by TEG skewed towards higher (Fig. 1). The value of ADP% was 80.11% ± 17.17% on average (ranging from 10.60 to 99.80%) in all study patients. According to the cut-off value of ADP% < 76% which defines the patients with HTPR under ticagrelor treatment, 45 patients (34.88%) had ADP% <76% and 84 patients (65.12%) had ADP% ≥76% in all enrolled patients.

**Fig. 2:**
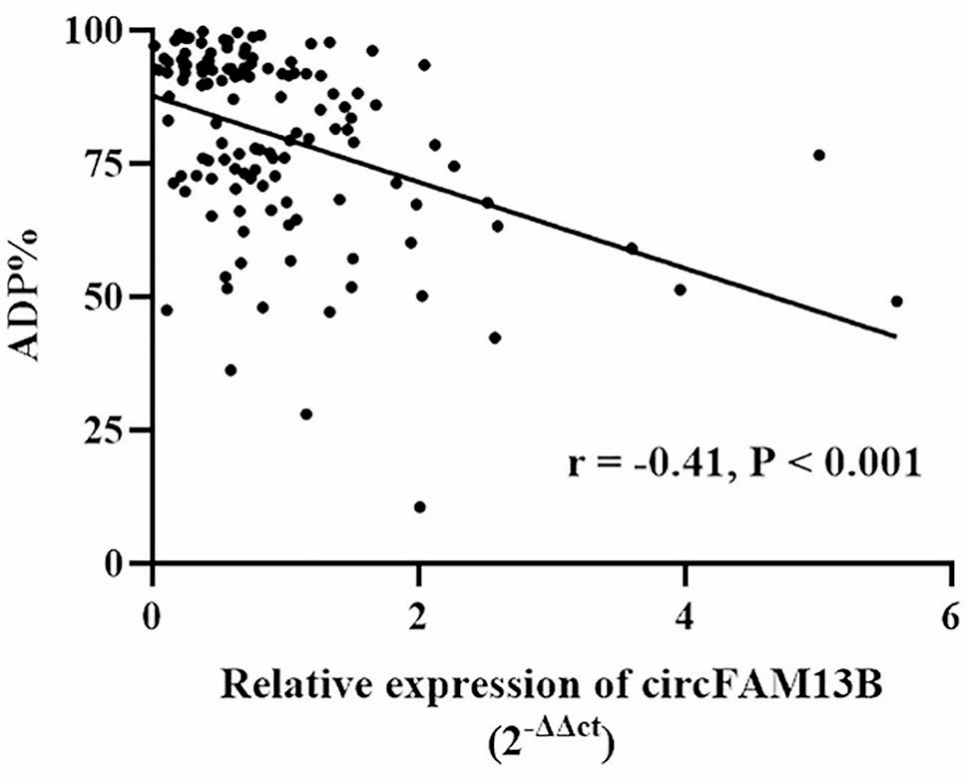
Correlation between the relative expression of platelet derived circFAM13B and ADP% measured by TEG in ticagrelor-treated patients with ACS. ADP%, adenosine diphosphate (ADP)-induced platelet inhibition rate; TEG, thromboelastography; ACS, acute coronary syndrome

### The expression of platelet-derived circFAM13B

To investigate the differential expression of circFAM13B in ACS patients treated with ticagrelor, platelet samples were obtained after ticagrelor treatment. The distribution of circFAM13B expression levels was measured by qRT-PCR, with an average expression level of 0.94 ± 0.88. The expression of circFAM13B derived from platelets was significantly higher in patients with ADP% < 76% who were treated with ticagrelor for ACS (1.27 ± 1.10 vs. 0.77 ± 0.67, *P* = 0.001).

### Association between the platelet-derived circFAM13B and ticagrelor antiplatelet responsiveness

**Fig. 3:**
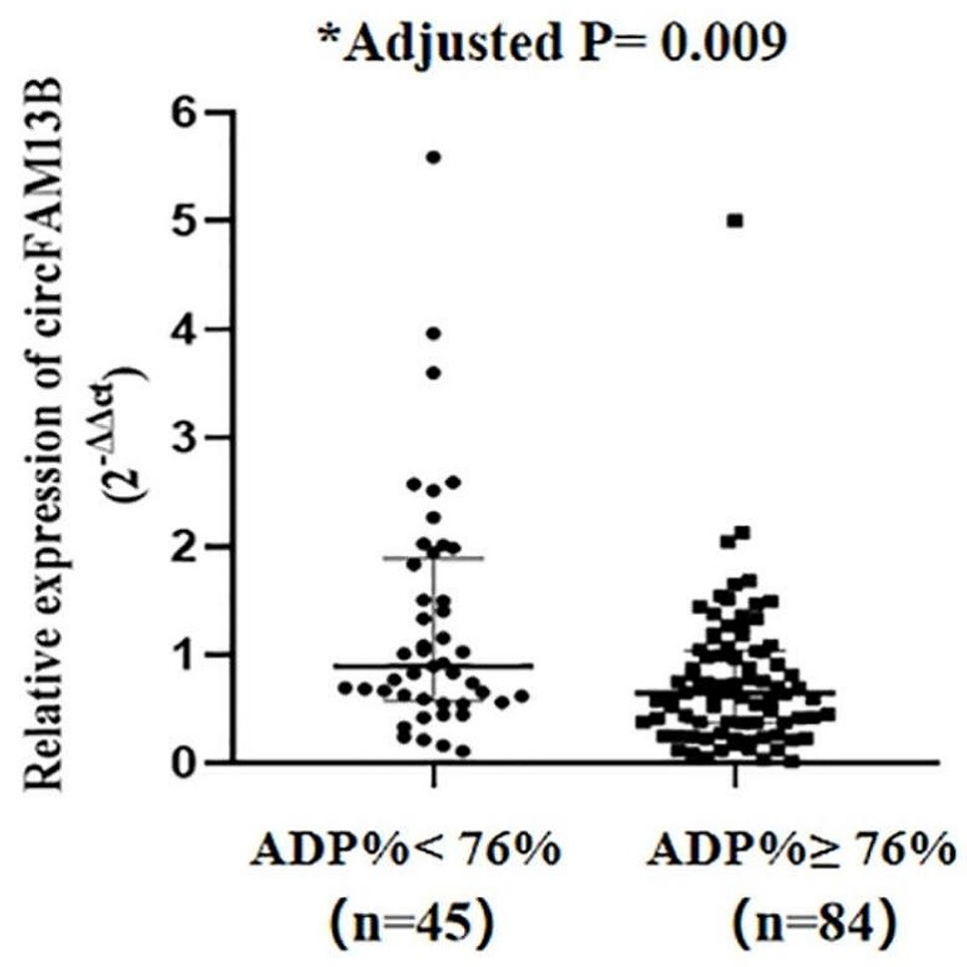
The expression of the platelet derived circFAM13B was presented relative to GAPDH based on double calculations of 2^−ΔΔCt^. The relative expression of platelet derived circFAM13B in ADP% value < 76% and ≥ 76% in ticagrelor treated patients with ACS. ^*^Adjusted by the covariates of age, smoking, history of stroke, 3-vessel diseases of coronary artery, diabetes mellitus and prior PCI. ADP%, adenosine diphosphate (ADP) induced platelet inhibition rate.

There is a negative correlation between circFAM13B levels and ADP% measured by TEG (*r* = -0.41, *P* < 0.001) (Fig. 2), and the expression of circFAM13B revealed a trend toward decline with increasing ADP% (Fig. 1). Compared to the patients with ADP% < 76%, those with ADP% ≥ 76% had significantly lower circFAM13B levels. In logistic regression analysis, we found that the platelet-derived circFAM13B was independently associated with ticagrelor antiplatelet responsiveness in ACS patients with the adjustment of clinical factors (including smoking, history of stroke, diabetes mellitus, prior PCI and 3-vessel diseases of the coronary artery) (*P* = 0.009, Fig. 3).

### Contribution of the platelet-derived circFAM13B to the prediction of adverse ischemic events

**Fig. 4:**
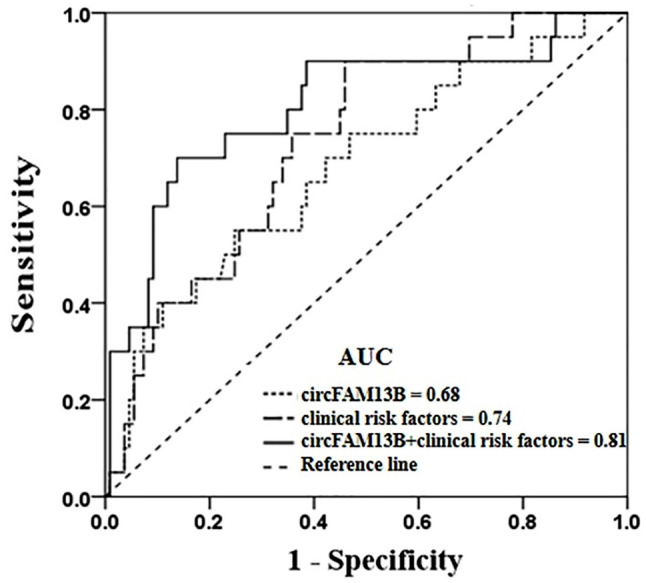
Receiver-operating characteristic curve for the prediction of adverse ischemic events in ticagrelor-treated patients with ACS according to the platelet derived circFAM13B with the relative expression level > 1.05 (dot line), clinical risk factors (solid and dot line), and the combination of circFAM13B and clinical risk factors (solid line). The clinical risk factors include age, smoking, history of stroke, 3-vessel diseases of coronary artery, diabetes mellitus, and prior PCI. AUC, area under the curve

**Fig. 5:**
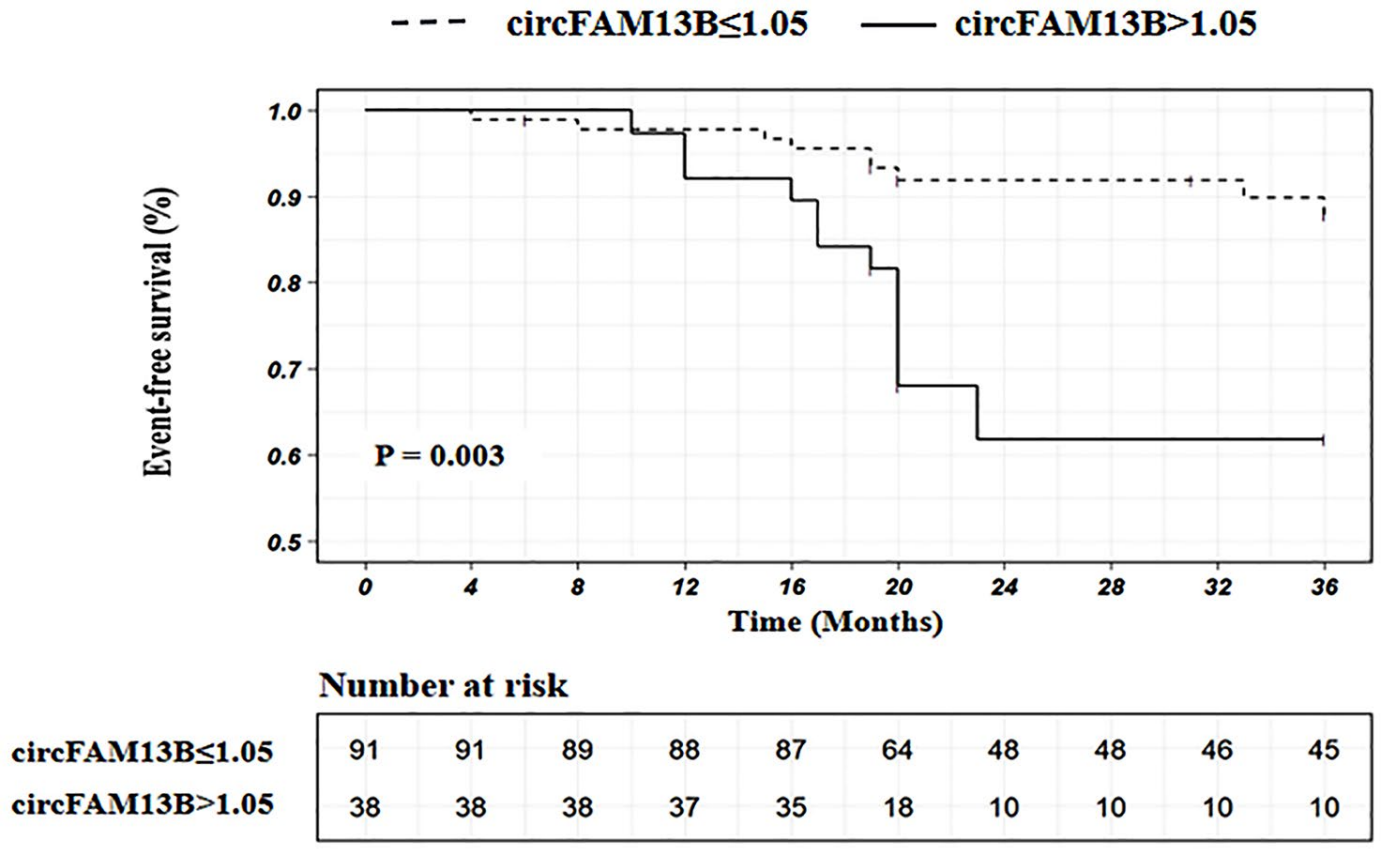
Kaplan–Meier survival curves for the adverse ischemic events within a follow-up of a median of 20 months in ticagrelor treated patients with ACS grouped according to the relative expression of platelet derived circFAM13B with the threshold of 1.05. ACS, acute coronary syndrome

Follow-up was available at a median of 20 months (interquartile range: 19 to 36 months). A total of 20 (15.50%) patients had adverse ischemic events, with 5 (3.88%) patients had adverse ischemic events within 1-year ticagrelor treatment, and 15 (11.63%) after 1 year. All-cause mortality was found in 2 patients (1.55%), nonfatal MI in 2 patients (1.55%), nonfatal stroke in 3 patients (2.32%), and rehospitalization for UA in 13 patients (10.08%). A ROC analysis showed that the expression levels of circFAM13B could distinguish the adverse ischemic events (AUC = 0.68, 95% CI: 0.55 to 0.81, *P* = 0.01) (Fig. 4). Overall, the relative expression of circFAM13B > 1.05 was identified as the optimal cut-off to predict the occurrence of the adverse ischemic events. We found that the cut-off value was an independent risk factor for the adverse ischemic events in both the univariate (unadjusted OR: 3.71, 95% CI: 1.39–9.91; *P* = 0.009) and the multivariate logistic regression analysis (adjusted OR: 5.60, 95% CI: 1.69–18.50; *P* = 0.005) (Table 2). Moreover, combining the cut-off value (relative expression of circFAM13B > 1.05) with clinical risk factors, including age, smoking, history of stroke, diabetes mellitus, prior PCI, and 3-vessel diseases of the coronary artery, improved the ability to predict the occurrence of the adverse ischemic events (AUC = 0.81, 95% CI: 0.69 to 0.92, *P* < 0.001) (Fig. 4) compared to clinical risk factors alone (AUC = 0.74, 95% CI: 0.63 to 0.84, *P* = 0.001) (Fig. 4). Kaplan-Meier curves for the adverse ischemic event were displayed in Fig. 5. On the basis of the cutoff value of circFAM13B > 1.05, the expression of circFAM13B could significantly separate patients at higher and lower risk for events mainly after 1 year (*p* = 0.003).

**Table 2:** The association between the platelet-derived expression of circFAM13B and clinical risk factors with adverse ischemic events in ticagrelor-treated patients with ACS

Variable	Univariate analysis			Multivariate analysis	
	OR (95% CI)	P Value		OR (95% CI)	P Value
Relative expression of circFAM13B > 1.05	3.71 (1.39–9.91)	**0.009**		5.60 (1.69–18.50)	**0.005**
Age	1.05 (0.98–1.12)	0.159		1.09 (1.01–1.18)	**0.033**
Smoking	1.14 (0.44–2.95)	0.792		2.20 (0.68–7.08)	0.186
History of stroke	0.82 (0.17–3.95)	0.805		0.51 (0.09–3.04)	0.459
Diabetes mellitus	2.21 (0.84–5.79)	0.108		3.64 (1.18–11.22)	**0.024**
Prior PCI	0.29 (0.08–1.06)	0.061		0.22 (0.05–0.94)	**0.041**
3-vessel diseases of coronary artery	0.70 (0.27–1.86)	0.479		0.77 (0.26–2.26)	0.636

Multivariate analysis was adjusted by age, smoking, history of stroke, diabetes mellitus, prior PCI and 3-vessel diseases of coronary artery. ACS, acute coronary syndrome; PCI, percutaneous coronary intervention. Adverse ischemic events were defined as all-cause death, nonfatal myocardial infarction, nonfatal stroke, stent thrombosis, and rehospitalization for unstable angina

## Discussion

The present study found that platelet-derived circFAM13B was correlated with the antiplatelet responsiveness and the occurrence of adverse ischemic events in ticagrelor treated patients with ACS. Therefore, circFAM13B might improve the ability to predict the occurrence of adverse ischemic events and could serve as one of the indicators to monitor antiplatelet treatment in patients with ACS. As far as we know, this is the first study to identify that platelet-derived circRNA could be informative for personalized antiplatelet therapy in patients with ACS.

Consistent with previous study [20], the variability of ADP-induced platelet aggregation could be observed in ACS patients receiving the ticagrelor treatment. A prospective study demonstrated that 46.2% of patients with ST-segment elevation myocardial infarction displayed HTPR after a 2-hour loading dose of ticagrelor [21]. The relatively lower incidence of HTPR in the present study might be primarily due to the different timing of the test measurements. Ischemic events occurred in ACS patients treated with ticagrelor, with the incidence rate ranging from 3.6 to 10.3 [6, 22–26]. The relatively higher incidence rate of ischemic events (15.5%) in the present study might be attributed to the inclusion of the rehospitalization for unstable angina. Our study aimed to explore whether platelet derived circFAM13B could affect the efficacy of the potent antithrombotic therapy of ticagrelor.

In the present study, we discovered that independent of the traditional clinical risk factors, platelet-derived circFAM13B was associated with antiplatelet efficacy of ticagrelor, with higher expression of circFAM13B in ticagrelor treated patients with HTPR. Moreover, the increased expression of circFAM13B in the present cohort conferred a 5-fold higher risk of clinical ischemic events. As shown in the Kaplan-Meier curves for the adverse ischemic event, the expression of circFAM13B could significantly separate patients at higher and lower risk for events, mainly after 12 months. Due to the extensive antiplatelet efficacy, the adverse ischemic events occurred only in 5 patients (3.88%) during 1-year ticagrelor treatment. However, residual risk of ischemic events occurred in 15 patients (11.63%) when most of the patients withdrew the ticagrelor treatment. The Kaplan-Meier survival analysis indicated that circFAM13B could predict not only the HTPR of ticagrelor, but also the occurrence of adverse events after 1-year ticagrelor treatment in patients with ACS. Therefore, it is more reasonable to conclude that platelet -derived circFAM13B could be predictive for the adverse ischemic events in ACS patients after the treatment with ticagrelor.

Several limitations should be mentioned. Firstly, the present study as a single-center with a relatively small sample size, may require further large-scale and multicenter studies to verify the association of circFAM13B with platelet reactivity and adverse ischemic events in ticagrelor treated patients with ACS. Secondly, we did not investigate the mechanism of circFAM13B affects the platelet reactivity. However, using a bioinformatics prediction analysis tool, we found that circFAM13B has the potential binding sites on miR-126 (http://mirwalk.umm.uni-heidelberg.de/) (data not shown). As a highly expressed miRNA in platelet, miR-126 has been discovered to be associated with platelet reactivity as well as the efficacy of antiplatelet therapy with the role on P2Y_12_ receptors, mainly through the regulation of the platelet surface proteins [27–29]. Therefore, we hypothesized that platelet-derived circFAM13B might functionally interact with miR-126 to influence the expression of P2Y_12_ receptor, the target of ticagrelor for antiplatelet treatment. Further basic research should be warranted to explore the potential mechanism. Thirdly, the study population in the present study was restricted to ACS patients treated with ticagrelor. Following studies required to encompass ACS patients treated with other P2Y_12_ receptor inhibitors (such as clopidogrel and prasugrel).

## Conclusion

Platelets-derived circFAM13B is associated with platelet reactivity and the occurrence of ischemic events in ACS patients treated with ticagrelor. Further research should be conducted to verify the mechanism of circFAM13B in the regulation of platelet reactivity and the antiplatelet responsiveness.

## Data Availability

The data used to support the findings of this study are available from the corresponding author (yintong301@163.com) upon request.

## Abbreviations

ACS: Acute coronary syndrome
ADP: Adenosine diphosphate
ADP%: ADP-induced platelet inhibition rate
AUC: Area under the curve
CircRNAs: Circular RNAs
DAPT: Dual antiplatelet therapy
HTPR: High on-treatment platelet reactivity
IQR: Interquartile range
MA: Maximum amplitude
MA_ADP_: ADP-induced platelet-fibrin clot strength
MA _fibrin_: Fibrin-induced clot strength
MA_Thrombin_: Thrombin-induced clot strength
MI: Myocardial infarction
NcRNA: Non-coding RNA
NSTEMI: Non-ST segment elevation myocardial infarction
PCI: Percutaneous coronary intervention
ROC: Receiver operating characteristic curve
STEMI: ST-segment elevation myocardial infarction
TEG: Thromboelastography
UA: Unstable angina

## References

[1] Byrne RA, Rossello X, Coughlan JJ, Barbato E, Berry C, Chieffo A, Claeys MJ, Dan GA, Dweck MR, Galbraith M (2023). 2023 ESC guidelines for the management of acute coronary syndromes. Eur Heart J.

[2] Sanderson NC, Parker WAE, Storey RF (2021). Ticagrelor: clinical development and future potential. Rev Cardiovasc Med.

[3] Husted S, Emanuelsson H, Heptinstall S, Sandset PM, Wickens M, Peters G (2006). Pharmacodynamics, pharmacokinetics, and safety of the oral reversible P2Y12 antagonist AZD6140 with aspirin in patients with atherosclerosis: a double-blind comparison to clopidogrel with aspirin. Eur Heart J.

[4] Wen M, Li Y, Qu X, Zhu Y, Tian L, Shen Z, Yang X, Shi X (2020). Comparison of platelet reactivity between prasugrel and ticagrelor in patients with acute coronary syndrome: a meta-analysis. BMC Cardiovasc Disord.

[5] Lemesle G, Schurtz G, Bauters C, Hamon M (2015). High on-treatment platelet reactivity with ticagrelor versus prasugrel: a systematic review and meta-analysis. J Thromb Haemost.

[6] Wallentin L, Becker RC, Budaj A, Cannon CP, Emanuelsson H, Held C, Horrow J, Husted S, James S, Katus H (2009). Ticagrelor versus clopidogrel in patients with acute coronary syndromes. N Engl J Med.

[7] Verdoia M, Sartori C, Pergolini P, Nardin M, Rolla R, Barbieri L, Schaffer A, Marino P, Bellomo G, Suryapranata H, De Luca G (2016). Prevalence and predictors of high-on treatment platelet reactivity with ticagrelor in ACS patients undergoing stent implantation. Vascul Pharmacol.

[8] Gutmann C, Joshi A, Zampetaki A, Mayr M (2021). The Landscape of Coding and noncoding RNAs in platelets. Antioxid Redox Signal.

[9] Inzulza-Tapia A, Alarcón M (2022). Role of non-coding RNA of human platelet in Cardiovascular Disease. Curr Med Chem.

[10] Alhasan AA, Izuogu OG, Al-Balool HH, Steyn JS, Evans A, Colzani M, Ghevaert C, Mountford JC, Marenah L, Elliott DJ (2016). Circular RNA enrichment in platelets is a signature of transcriptome degradation. Blood.

[11] Preußer C, Hung LH, Schneider T, Schreiner S, Hardt M, Moebus A, Santoso S, Bindereif A (2018). Selective release of circRNAs in platelet-derived extracellular vesicles. J Extracell Vesicles.

[12] Li X, Yang L, Chen LL (2018). The Biogenesis, functions, and challenges of Circular RNAs. Mol Cell.

[13] The database is freely accessible. through the web server at http://circinteractome.nia.nih.gov.Accessed 11 Nov. 2023.

[14] Lv J, Li K, Yu H, Han J, Zhuang J, Yu R, Cheng Y, Song Q, Bai K, Cao Q (2023). HNRNPL induced circFAM13B increased bladder cancer immunotherapy sensitivity via inhibiting glycolysis through IGF2BP1/PKM2 pathway. J Exp Clin Cancer Res.

[15] Ning L, Long B, Zhang W, Yu M, Wang S, Cao D, Yang J, Shen K, Huang Y, Lang J (2018). Circular RNA profiling reveals circEXOC6B and circN4BP2L2 as novel prognostic biomarkers in epithelial ovarian cancer. Int J Oncol.

[16] Xie Y, Hang X, Xu W, Gu J, Zhang Y, Wang J, Zhang X, Cao X, Zhan J, Wang J, Gan J (2021). CircFAM13B promotes the proliferation of hepatocellular carcinoma by sponging miR-212, upregulating E2F5 expression and activating the P53 pathway. Cancer Cell Int.

[17] Zou Y, Wang Y, Wu Y, Zhang S, Liu H, Yin T (2022). Prediction of residual ischemic risk in ticagrelor-treated patients with acute coronary syndrome. Thromb J.

[18] Gurbel PA, Bliden KP, Navickas IA, Mahla E, Dichiara J, Suarez TA, Antonino MJ, Tantry US, Cohen E (2010). Adenosine diphosphate-induced platelet-fibrin clot strength: a new thrombelastographic indicator of long-term poststenting ischemic events. Am Heart J.

[19] Bliden KP, DiChiara J, Tantry US, Bassi AK, Chaganti SK, Gurbel PA (2007). Increased risk in patients with high platelet aggregation receiving chronic clopidogrel therapy undergoing percutaneous coronary intervention: is the current antiplatelet therapy adequate?. J Am Coll Cardiol.

[20] Siller-Matula JM, Akca B, Neunteufl T, Maurer G, Lang IM, Kreiner G, Berger R, Delle-Karth G (2016). Inter-patient variability of platelet reactivity in patients treated with prasugrel and ticagrelor. Platelets.

[21] Alexopoulos D, Xanthopoulou I, Gkizas V, Kassimis G, Theodoropoulos KC, Makris G, Koutsogiannis N, Damelou A, Tsigkas G, Davlouros P, Hahalis G (2012). Randomized assessment of ticagrelor versus prasugrel antiplatelet effects in patients with ST-segment-elevation myocardial infarction. Circ Cardiovasc Interv.

[22] Schüpke S, Neumann FJ, Menichelli M, Mayer K, Bernlochner I, Wöhrle J, Richardt G, Liebetrau C, Witzenbichler B, Antoniucci D (2019). Ticagrelor or Prasugrel in patients with Acute Coronary syndromes. N Engl J Med.

[23] Turgeon RD, Koshman SL, Youngson E, Har B, Wilton SB, James MT, Graham MM (2020). Association of Ticagrelor vs clopidogrel with major adverse coronary events in patients with acute coronary syndrome undergoing percutaneous coronary intervention. JAMA Intern Med.

[24] Larmore C, Effron MB, Molife C, DeKoven M, Zhu Y, Lu J, Karkare S, Lieu HD, Lee WC, Vetrovec GW (2016). Real-world comparison of prasugrel with ticagrelor in patients with acute coronary syndrome treated with percutaneous coronary intervention in the United States. Catheter Cardiovasc Interv.

[25] Effron MB, Nair KV, Molife C, Keller SY, Page RL, Simeone JC, Murphy B, Nordstrom BL, Zhu Y, McCollam PL (2018). Vetrovec GW: one-year clinical effectiveness comparison of prasugrel with ticagrelor: results from a retrospective observational study using an integrated claims database. Am J Cardiovasc Drugs.

[26] Han Y, Claessen BE, Chen SL, Chunguang Q, Zhou Y, Xu Y, Hailong L, Chen J, Qiang W, Zhang R (2022). Ticagrelor with or without aspirin in Chinese patients undergoing percutaneous coronary intervention: a twilight China substudy. Circ Cardiovasc Interv.

[27] Krammer TL, Mayr M, Hackl M. microRNAs as promising biomarkers of platelet activity in antiplatelet therapy monitoring. Int J Mol Sci 2020, 21. 10.3390/ijms21103477 PMC7278969 32423125

[28] Jansen F, Yang X, Proebsting S, Hoelscher M, Przybilla D, Baumann K, Schmitz T, Dolf A, Endl E, Franklin BS (2014). MicroRNA expression in circulating microvesicles predicts cardiovascular events in patients with coronary artery disease. J Am Heart Assoc.

[29] Czajka P, Fitas A, Jakubik D, Eyileten C, Gasecka A, Wicik Z, Siller-Matula JM, Filipiak KJ, Postula M (2021). MicroRNA as potential biomarkers of platelet function on antiplatelet therapy: a review. Front Physiol.

